# Institutional Notes and News

**Published:** 1911-01-14

**Authors:** 


### Institutional Notes and News.

**Royal Infirmary, Newcastle.**

As foreshadowed in a previous issue, the year was not allowed to come to an end at Newcastle without the conference between the representatives of the house committee and the honorary medical staff of Newcastle Royal Infirmary having settled the dispute which caused the recent resignation of the medical staff. The whole matter was dealt with so fully in a special article which we published on December 24, page 385, that it is unnecessary to add further comment here. At the conference it was agreed to withdraw those portions of the report of the Admissions Committee, which referred to undue preference being shown in the admission of patients, to which the staff had objected. The result of the conference will be presented to the House Committee at their next meeting, when the resignations most probably will be withdrawn.

**Royal Infirmary, Edinburgh.**

The Royal Infirmary of Edinburgh has concluded a busy year's work, for its latest annual report records the admission of 12,342 cases to the wards and of 38,930 out-patients. The daily average number of patients in the hospital was 842, and as each of these was entitled to two visitors' tickets the traffic in the building was increased to an extent that was found in need of correction. A special committee therefore has been sitting, and having discovered that the old allowance of visiting twice a day to country patients and four times a week to town patients was much more extensive than that allowed at other hospitals, has proposed, among other things: (1) That the morning visiting hour (9 to 10 a.m) be abolished; (2) that, owing to the impossibility of distinguishing between town and country friends, the visiting days and hours be made equal for both, and only one form of ticket be used; and (3) that these visiting days and hours be as follows: Sundays, from 4 to 5 p.m.; Wednesdays, from 3 to 4 p.m.; and Tuesdays, Thursdays, and Saturdays from 5 to 6 p.m The morning visiting hour was originally introduced for country visitors. Experience has shown, however, that it is almost exclusively taken advantage of by the city friends of country patients, and is, therefore, not really required. The total ordinary income of the hospital was £36,129, and the ordinary expenditure £54,217.

**Addenbrooke's Hospital, Cambridge.**

A busy and energetic year's work is summarised in the annual report of Addenbrooke's Hospital, Cambridge, and, what is more, this summary is attractively presented and made as readable as good printing, paper, and a generous supply of photographs can make it. There is nothing depressing about the cover, and, when that is turned, a variety of interesting local information is to be found which gives quite a comprehensive idea of the activities, the hopes and fears of the institution. Two facts stand out from the report, however, the first of which is the great increase in the number of out-patients, 9,733 having been received, as compared with 8,347 in 1909. The second is the creditable way in which a balance on the right side continues to appear, in spite of the increased expenses due to increased numbers. Of the nature of these expenses it is only necessary to add that a new out-patient department is now made a necessity, and we were glad to record, in The Hospital of November 5, page 181, that its provision, together with that of a new children's ward, had been adopted by the town as the local memorial to King Edward. A sum of £15,000 is required for this purpose, of which more than half has been already subscribed. This is typical of the good relations of the University town and its hospital to one another, for it is these alone which can ensure the continued solvency of Addenbrooke's as new demands are made upon it. Of course efforts have been made to find new sources of income, and perhaps the most striking of these was the foundation of the Cambridge and District Workers' Hospital Fund in the summer. For details of the successful launching of this our readers are referred to previous issues, those of July 30 and October 22 in particular, where its foundation, aims and first quarter's progress were described in full. This fund promises to develop extensively, and Mr. Richard J. Coles, the superintendent, is to be congratulated on the success of Mr. J. E. Papworth's efforts as its secretary, since some certain source of income must be found when legacies are not multitudinous. Two, amounting to under £200, were received last year by Addenbrooke's. This report should confirm and strengthen the goodwill of every subscriber who has the good fortune to read it.

**Wimbledon Hospital.**

The committee of management of the Wimbledon Hospital has decided to rebuild the institution, and there is little doubt that the subscription of £550 which King Edward's Hospital Fund has promised will influence the general public to give too. This is important, as an old friend has offered to hand over £1,000 to the hospital provided that an equal sum shall be raised before the end of this month. The first part of the new building will consist of the administrative block and two wings, and in one of the latter will be the "King Edward VII. Ward" as a memorial to the late King. Another memorial ward will be the "Collyns Ward," in commemoration of Dr. Poole-Collyns, the first medical officer of the hospital, who died a few years ago. For this over £900 has been subscribed. But in order to complete the new hospital free of debt an appeal is being made for £3,000. We hope this quickly will be forthcoming; at any rate, it is certain that a King Edward VII. ward is regarded by the public as a whole with more favour than any other form of memorial to the late King.

**Chelsea Hospital for Women.**

The annual entertainment for the patients and nurses organised by the Ladies' Committee of the Chelsea Hospital for Women, Fulham Road, S.W., was a great success. Viscountess Esher, the vice-president, presided. After Mr. Lambert's conjuring, there was the distribution of a Christmas-tree laden with presents, which were the gift of Mr. Fenton, senior surgeon, and Mrs. Fenton. The work in the wards of the hospital during the past year shows a marked increase, the number of admissions being 870, as compared with 803 for 1909. The reception of diseases peculiar to women, the provision made for those of small means, and the privacy of treatment afforded, are the special features which mark this hospital. The cure of the patients, moreover, is consolidated at its convalescent home at St. Leonards-on-Sea. The treasurer, Mr. Henry E. Wright, or the secretary, Mr. Herbert H. Jennings, would gratefully welcome new annual subscriptions.

